# Assembly of a novel biosynthetic pathway for gentamicin B production in *Micromonospora echinospora*

**DOI:** 10.1186/s12934-015-0402-6

**Published:** 2016-01-05

**Authors:** Xianpu Ni, Zhenpeng Sun, Yawen Gu, Hao Cui, Huanzhang Xia

**Affiliations:** School of Life Science and Biopharmaceutics, Shenyang Pharmaceutical University, No.103 Wenhua Road, Shenyang, Liaoning China

**Keywords:** *Micromonospora echinospora*, Gentamicin B, Metabolic engineering, Artificial biosynthetic pathway

## Abstract

**Background:**

Isepamicin is a weakly toxic but highly active aminoglycoside antibiotic derivative of gentamicin B. Gentamicin B is a naturally occurring minor component isolated from *Micromonospora echinospora*. 2ʹ-NH_2_-containing gentamicin C complex is a dominant compound produced by wild-type *M*. *echinospora*; by contrast, 2ʹ-OH-containing gentamicin B is produced as a minor component. However, the biosynthetic pathway of gentamicin B remains unclear. Considering that gentamicin B shares a unique C_2ʹ_ hydroxyl group with kanamycin A, we aimed to design a new biosynthetic pathway of gentamicin B by combining twelve steps of gentamicin biosynthesis and two steps of kanamycin biosynthesis.

**Results:**

We blocked the biosynthetic pathway of byproducts and generated a strain overproducing JI-20A, which was used as a precursor of gentamicin B biosynthesis, by disrupting *gen*K and *gen*P. The amount of JI-20A produced in *M. echinospora* ∆K∆P reached 911 μg/ml, which was 14-fold higher than that of *M. echinospora* ∆P. The enzymes KanJ and KanK necessary to convert 2ʹ-NH_2_ into 2ʹ-OH from the kanamycin biosynthetic pathway were heterologously expressed in *M. echinospora* ΔKΔP to transform JI-20A into gentamicin B. The strain with *kan*JK under PermE* could produce 80 μg/ml of gentamicin B, which was tenfold higher than that of the wild-type strain. To enhance gentamicin B production, we employed different promoters and gene integration combinations. When a P*hrd*B promoter was used and *kan*J and *kan*K were integrated in the genome through gene replacement, gentamicin B was generated as the major product with a maximum yield of 880 μg/ml.

**Conclusion:**

We constructed a new biosynthetic pathway of high-level gentamicin B production; in this pathway, most byproducts were removed. This method also provided novel insights into the biosynthesis of secondary metabolites via the combinatorial biosynthesis.

**Electronic supplementary material:**

The online version of this article (doi:10.1186/s12934-015-0402-6) contains supplementary material, which is available to authorized users.

## Background

Aminoglycoside antibiotics have been widely used to treat severe bacterial infections; for instance, streptomycin was administered as the first effective anti-tuberculosis agent. Other aminoglycoside antibiotics include 2-deoxystreptamine (2-DOS) containing gentamicin, kanamycin, neomycin, and butirosin. However, the critical resistance mechanism of aminoglycoside antibiotics in pathogens is enzymatic inactivation. As such, semi-synthetic aminoglycosides have been created to overcome pathogen enzymatic inactivation. For example, isepamicin is developed by introducing (S)-3-amino-2-hydroxypropionyl side chains to the 1-amino group of gentamicin B. This side chain can block the modification by various aminoglycoside-modifying enzymes. Thus, isepamicin is highly stable against aminoglycoside-inactivating enzymes [1].

Isepamicin is manufactured from gentamicin B, which is co-produced in *Micromonospora echinospora*. Gentamicin C complex is produced by *M. echinospora* as its major product. By contrast, gentamicin A, B, and X are yielded by *M. echinospora* as minor components [2]. Gentamicin A and X are intermediates of gentamicin C biosynthesis. However, gentamicin B is not an intermediate of the gentamicin C biosynthetic pathway because gentamicin B cannot be biotransformed by *M. echinospora* into any gentamicin C component [2]. Thus far, the biosynthetic pathway of gentamicin B remains unclear.

**Fig. 1:**
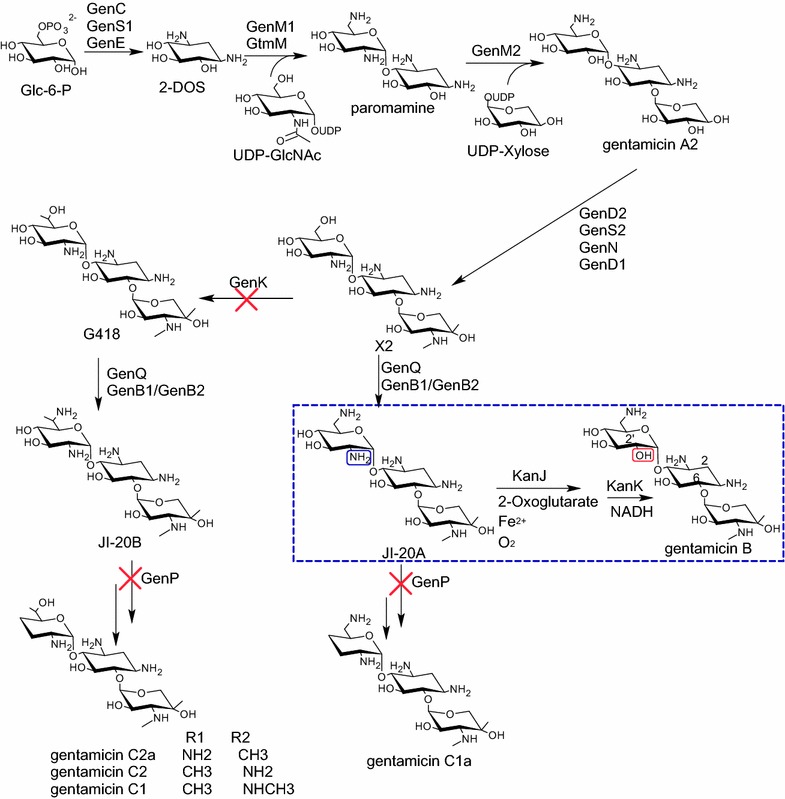
Biosynthetic pathway for gentamicin and the proposed strategy for gentamicin B biosynthetic pathway (inside the *dashed line*)

The biosynthetic pathway of gentamicin has been elucidated (Fig. 1). The gene cluster of gentamicin has also been cloned [3, 4]. The genes involved in the biosynthesis of pseudodisaccharide paromamine and pseudotrisaccharide gentamicin A2 have been identified [5]. The production of gentamicin X2 from A2 involves four enzymes: oxidoreductase, transaminase, and two methyltransferases [6]. X2 can also be transformed into JI-20A by C-6ʹ dehydrogenase and transaminase [7–9]. Moreover, GenK is the methyltransferase of C-6ʹ in gentamicin, and *gen*K disruption generates a gentamicin C1a-overproducing strain [10–12]. GenP is a phosphotransferase participating in 3ʹ,4ʹ-deoxygenation in the gentamicin biosynthetic pathway [13]. If *gen*P is disrupted, JI-20A,JI-20B, and JI-20Ba accumulate in mutant strains [9]. In this study, *gen*K and *gen*P were disrupted simultaneously to generate strains producing JI-20A, which was designed as a precursor of gentamicin B biosynthesis (Fig. 1).

The structure of gentamicin B resembles that of both gentamicin C and kanamycin A. Unlike most 2-DOS-containing aminoglycosides, kanamycin A contains a hydroxy group at C_2ʹ_; gentamicin B shares this unique structure with kanamycin A. The methylated pentose ring of gentamicin B is also similar to that of gentamicin C and is designated as garaosamine. Gentamicin B can be synthesized via a biosynthetic pathway similar to that of kanamycin A on the basis of molecular structure. In the kanamycin A biosynthetic pathway in *Streptomyces kanamyceticus*, 2ʹ-NH_2_ is converted into 2ʹ-OH by using KanJ and KanK; in the process, 2-oxoglutarate, Fe^2+^, O_2_, and NADH are utilized [14]. We hypothesized that gentamicin B could be biosynthesized from JI-20A, a compound structurally similar to gentamicin B, except NH_2_ group at C_2ʹ_ (Fig. 1). Thus, JI-20A could be converted into gentamicin B in JI-20A-overproducing strain via two steps catalyzed by KanJ and KanK. Using the assembly and metabolically engineered biosynthesis pathway of gentamicin B, we can eliminate byproducts and improve gentamicin B production.

## Results and discussion

### Construction of JI-20A-overproducing strain by disrupting *gen*P and *gen*K genes

The original strain can potentially generate gentamicin B; as such, we determined whether gentamicin B is synthesized from the 2ʹ-amino-containing precursor by the KanJ and KanK homologs. However, the genes homologous to *kan*J and *kan*K have yet to be detected in *M. echinospora*. Considering that the biosynthetic pathway of gentamicin B remains unclear, we aimed to construct an artificial pathway of gentamicin B biosynthesis. JI-20A and gentamicin B are similar in terms of chemical structure except at C2ʹ. JI-20A contains an amino group at C2ʹ, whereas gentamicin B comprises a hydroxyl group at the same position. The structural difference between JI-20A and gentamicin B is similar to the difference between kanamycin A and B. Considering that kanamycin B is converted into kanamycin A by KanJ and KanK, we determined whether KanJ and KanK can also transform JI-20A into gentamicin B.

**Fig. 2:**
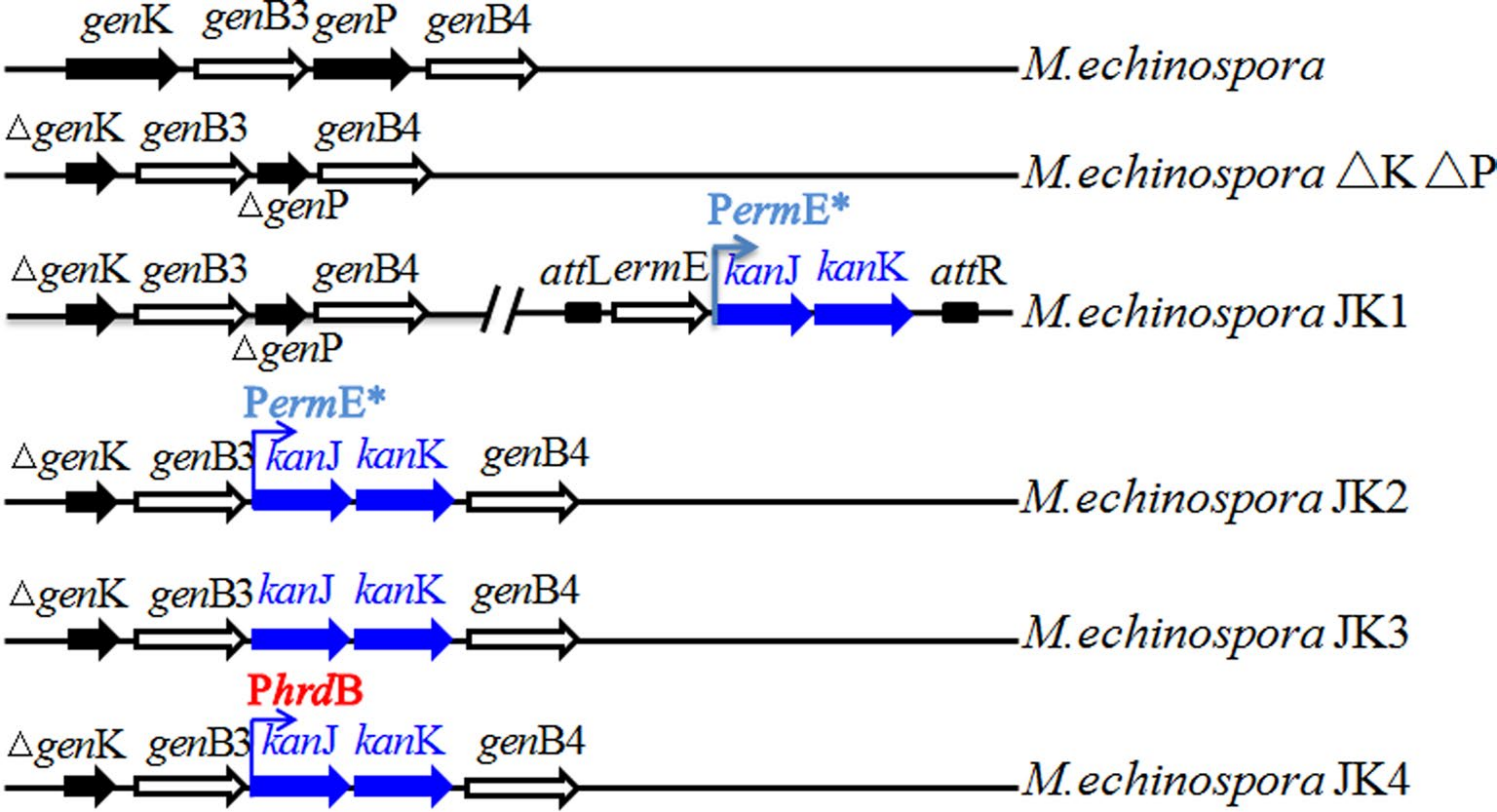
Genotypes of *M. echinospora* and its recombinant strains

**Fig. 3:**
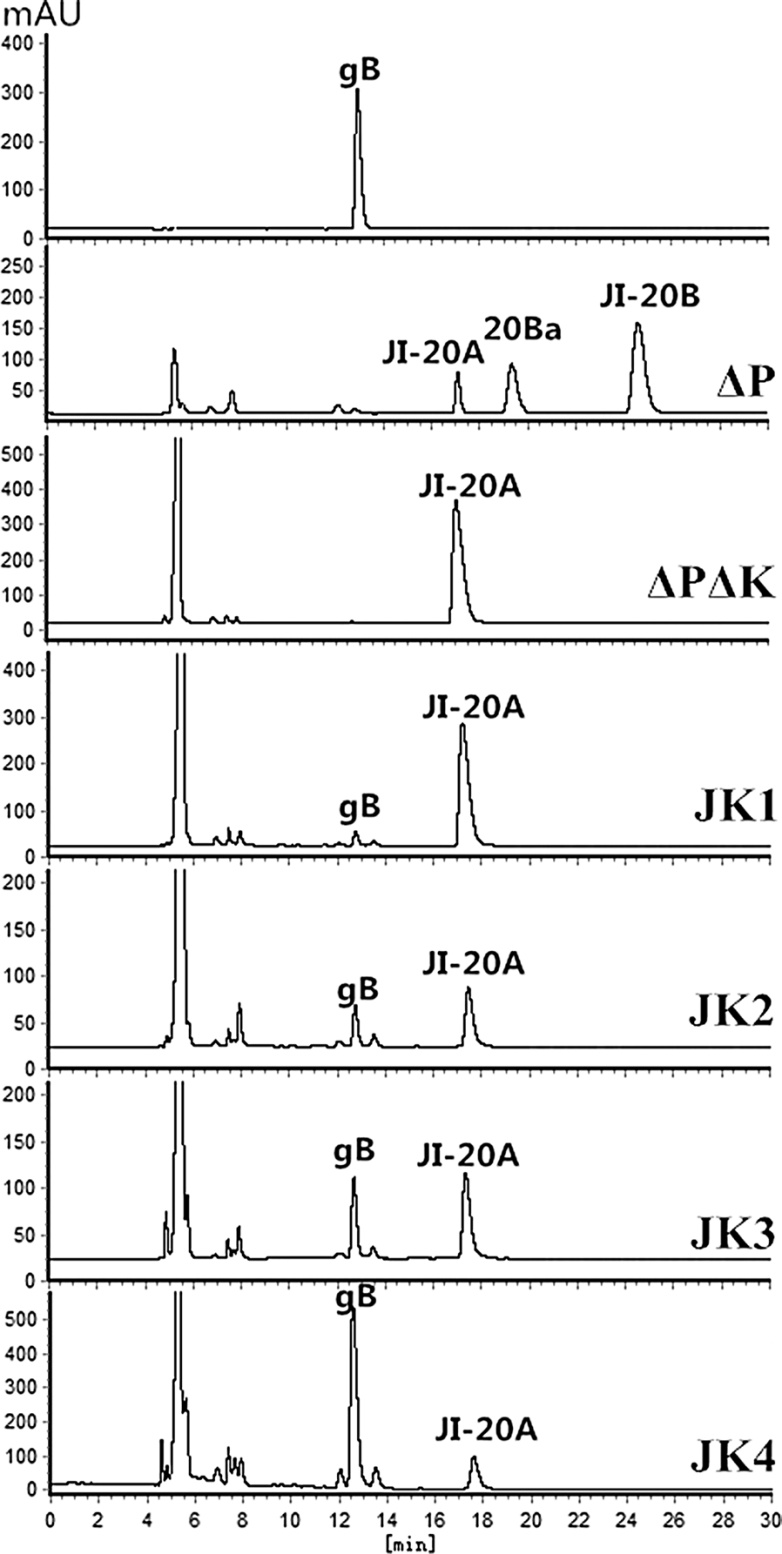
HPLC-ELSD analysis of gentamicin production in fermentations. *gB* gentamicin B, *20Ba* JI-20Ba

To generate a JI-20A-producing strain, we simultaneously disrupted the 6ʹ-methyltransferase gene *gen*K and the 3ʹ-phosphotransferase gene *gen*P in this study (Fig. 2). We separately generated *gen*P- and *gen*K-disrupting strains in our previous work [6, 7]. The *gen*P-disrupting strain does not produce gentamicin C complex, which are the main products of the wild-type strain but are the main byproducts in gentamicin B production. In this study, the *gen*K-disrupting plasmid was introduced to *M. echinospora* ∆P to obtain the double-gene-disrupting strain *M. echinospora* ∆K∆P (Additional file 1: Figure S1A). The disrupting strain was fermented and its products were analyzed through high-performance liquid chromatography with evaporative light scattering detector (HPLC-ELSD). JI-20B and JI-20Ba were the main products of *M. echinospora* ∆P but were undetectable in *M. echinospora* ∆K∆P (Fig. 3). JI-20A was produced up to 911 μg/ml, which was 14-fold higher than that produced by *M. echinospora* ∆P. We blocked the biosynthesis of gentamicin C1, C1a, C2, C2a, JI-20B, and JI-20Ba and generated a JI-20A-overproducing strain by disrupting *gen*P and *gen*K.

### Construction of a new gentamicin B biosynthetic pathway through the heterologous expression of *kan*J and *kan*K

The *kan*J and *kan*K genes under the control of the strong promoter P*erm*E* were cloned into the site-specific integration plasmid pEAP1 to construct pSPUJK1. pSPUJK1 was introduced to *M. echinospora* ∆K∆P through conjugation and exconjugants were selected through erythromycin resistance; as a result, *M. echinospora* JK1 was generated (Additional file 1: Figure S1B). *M. echinospora* JK1 was fermented under the same condition used to ferment the wild-type strain. The fermentation products of *M. echinospora* JK1 were also analyzed through HPLC-ELSD. Compared with the wild-type strain, *M. echinospora* JK1 produced a new product exhibiting a retention time similar to that of gentamicin B (Fig. 3).

**Fig. 4:**
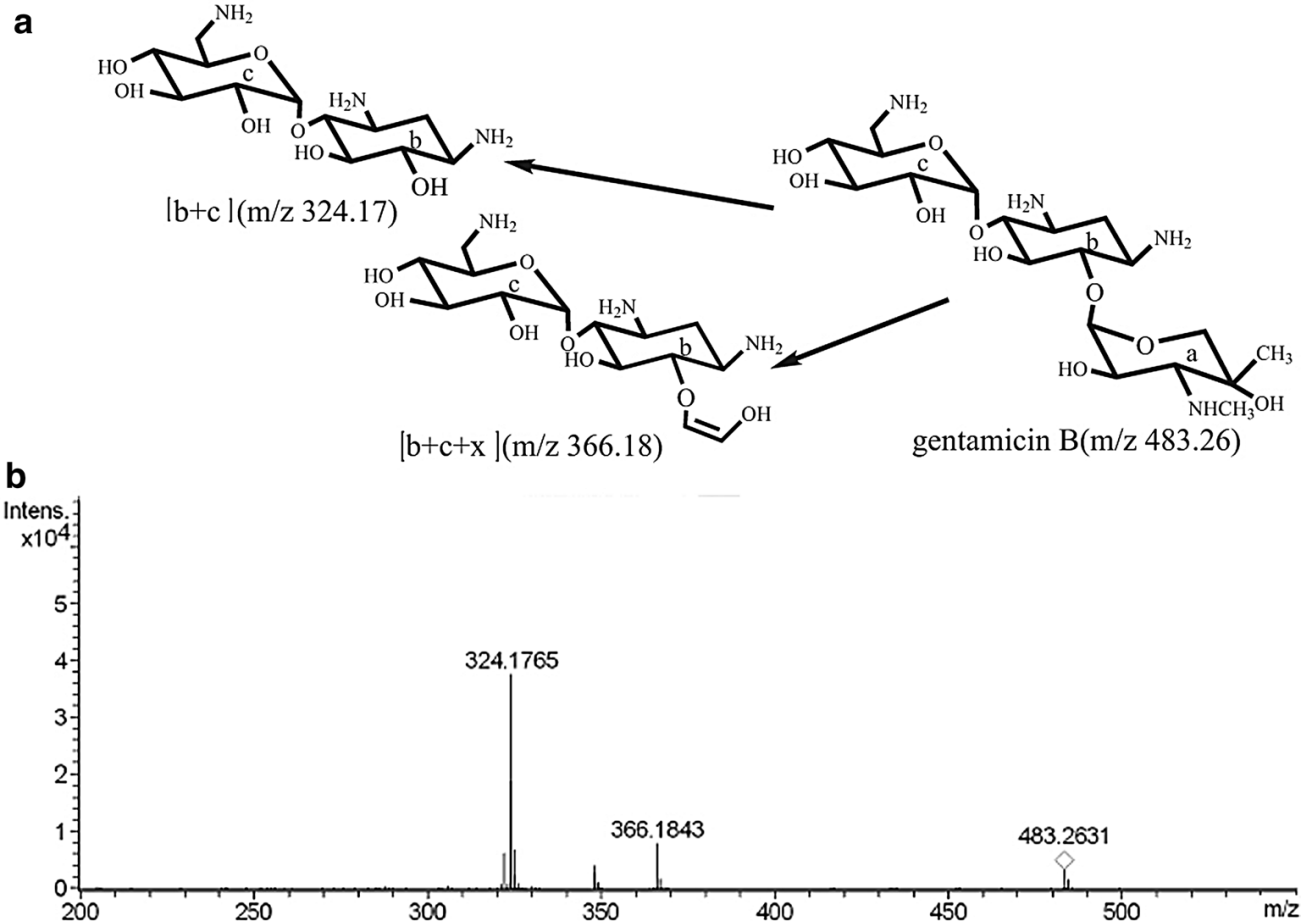
Structural determination of gentamicin B by MS/MS. **a** Structure of gentamicin B. **b** Mass spectra of the new product from *M. echinospora*JK1

To determine the structure of the new product of *M. echinospora* JK1, we analyzed the purified product through mass spectrometry. The exact mass of the product was 482.26 (*m*/*z* 483.26), which corresponds to gentamicin B (Fig. 4). Furthermore, the protonated fragments were the same as those of the reported mass spectra of gentamicin B [15], that is, the glycoside bond of gentamicin B cleavage formed the fragment b + c (*m*/*z* 324.17). The glycoside found at the C_6_–O of 2-DOS decomposed; thus, the fragments b + c + x (*m*/*z* 366.18) were produced. Nevertheless, gentamicin B yields the same molecular weight as that of gentamicin X2, which is another minor component of gentamicin. To verify the structure of the new product, we recorded the corresponding ^1^H and ^13^C NMR data. The ^1^H spectrum of the new product was similar to that of gentamicin B [16] (Additional file 2: Figure S2). Moreover, the ^13^C NMR data of the new product (Table 1) were identical with the reported NMR data of gentamicin B [17]. MS and NMR data demonstrated that the product from *M. echinospora* JK1 is gentamicin B.

**Table 1:** ^13^C NMR spectral data for gentamicin B [16] and the new compound from *M. echinospora* JK1 (for atom numbering see Fig. 1)

Atom	Gentamicin B (δ)	New compound (δ)
1	50.6	49.7
2	28.3	27.5
3	48.4	47.5
4	79.0	78.3
5	73.1	72.3
6	84.6	83.8
1′	96.6	95.9
2′	71.7	70.6
3′	73.0	72.0
4′	71.6	69.8
5′	69.5	68.6
6′	41.1	40.1
1′′	102.0	101.2
2′′	67.2	67.6
3′′	64.2	63.3
4′′	70.8	70.6
5′′	68.6	66.2
3′′–N–CH_3_	35.3	34.3
4′′–CH_3_	21.7	20.8

Although gentamicin B production is greatly improved in *M. echinospora* JK1 compared with that of the wild-type strain, the yield is only 85 μg/ml in *M. echinospora* JK1. A large quantity of JI-20A in *M. echinospora* JK1 was also found in the fermentation broth. Therefore, the expression levels of *kan*J and *kan*K were genetically manipulated to improve gentamicin B production.

### Enhancing gentamicin B production through the genetic manipulation of *kan*JK

**Fig. 5:**
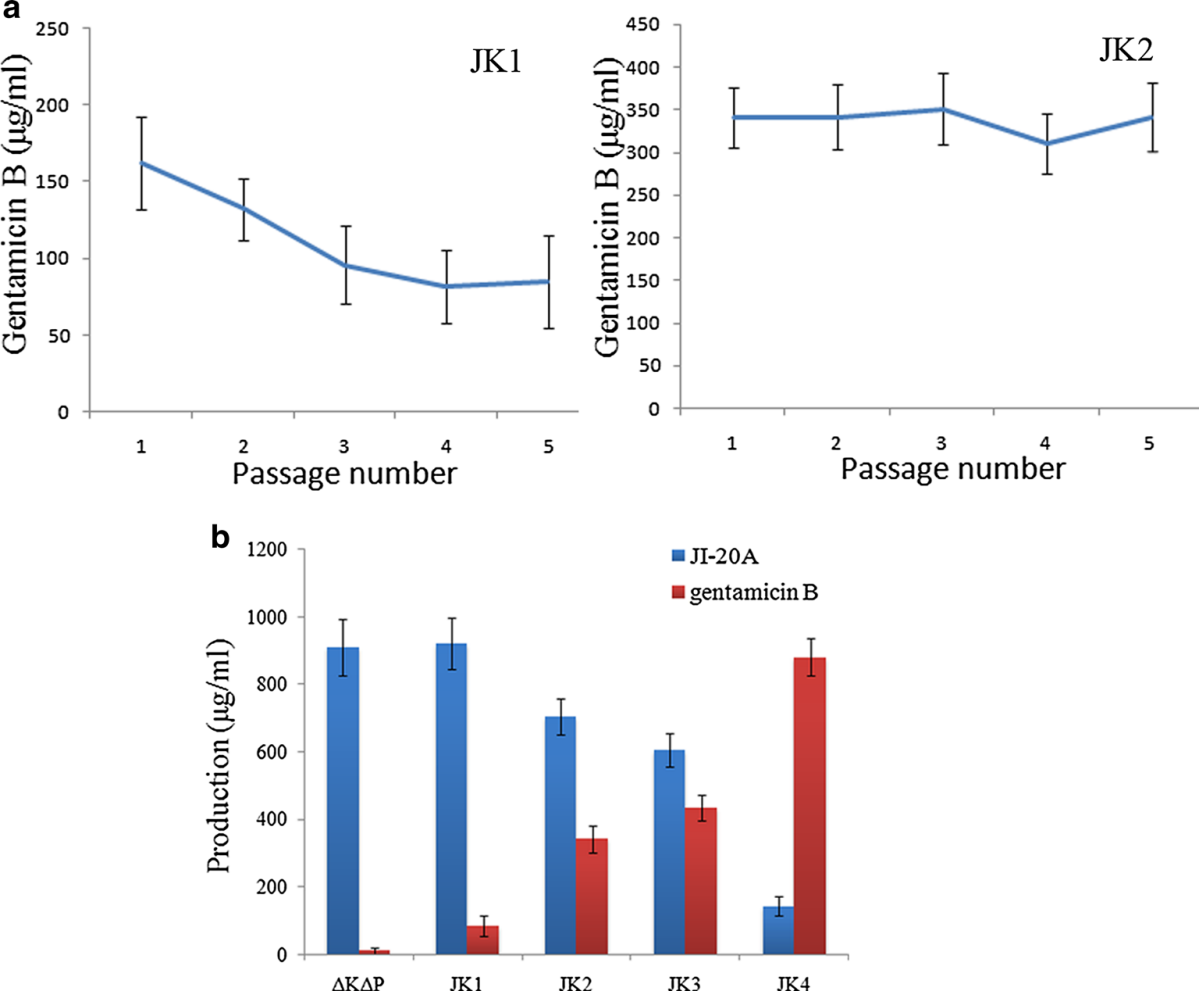
Production and stability analysis of gentamicin B-producing strains. **a** Stability of gentamicin B production in *M. echinospora*JK1 and JK2 strains. **b** Production analysis of gentamicin B in *M. echinospora* ΔKΔP and *kan*JK heterologous expression strains. Error bars represent standard deviations. Samples were analyzed through HPLC and quantified on the basis of the peak areas

The genetic stability of the strains applied in industrial fermentation is very important. However, gentamicin B production in *M. echinospora* JK1 decreased from 162 μg/ml to 80 μg/ml in five generations of unselected passages (Fig. 5a). We proposed that the instability of *M. echinospora* JK1 is caused by the homologous recombination between the P*erm*E* upstream of *kan*JK and the native promoter of *erm*E, which was used as a selection marker gene in pSPUJK1. In addition, the instability may be caused by chromosomal rearrangements or plasmid elimination from the chromosome, which can occur when a φC31-derived plasmid is used in strains containing multiple pseudo *att*B sites [18].

To avoid genetic instability, we integrated *kan*J and *kan*K into the chromosome through homologous recombination instead of site-specific insertion. Considering that *gen*P has been disrupted in *M. echinospora* ∆K∆P, we designed *kan*J and *kan*K to replace *gen*P (Additional file 1: Figure S1C). To reduce JI-20A production and improve gentamicin B production, we placed *kan*J and *kan*K under the control of the strong promoter P*erm*E* (Fig. 2). Moreover, the fermentation broth of the gene replacement strain *M. echinospora* JK2 was analyzed through HPLC-ELSD. Figure 5a shows that the gentamicin B production by *M. echinospora* JK2 was more stable than that by *M. echinospora* JK1. Gentamicin B production increased to 342 μg/ml. However, *M. echinospora* JK2 produced a considerable quantity of JI-20A.

The native promoter of *gen*P was used to improve gentamicin B production. To eliminate any possibility of polar effects on other genes, we designed *kan*JK as a replacement of the open reading frame of *gen*P, and no other elements were introduced (Additional file 1: Figure S1D). As such, the native ribosome binding site (RBS) and promoter of *gen*P (or promoter of its operon) were employed by *kan*JK. Although the regulatory elements of *gen*P have yet to be determined, we proposed that all regulatory elements of *gen*P can be used. In the fermentation of *M. echinospora* JK3, *kan*JK was expressed as gentamicin B was produced in JK3. Gentamicin B production also reached 436 μg/ml. P*erm*E*, which exhibits a strong promoter activity, is widely used for the gene expression in *Actinomyces*. However, this work found that P*erm*E* is weaker than the native promoter of *gen*P.This phenomenon occurred possibly because P*erm*E* originates from *Saccharopolyspora erythraea*; as such, P*erm*E* cannot be as effective in *M. echinospora* as in *S. erythraea*. Another possible cause is the ineffectiveness of the RBS of the construction because the translation initiation of P*erm*E*-*kan*J-*kan*K is weak when analyzed with the RBS Calculator [19].

JI-20A accumulation in the fermentation broths of *M. echinospora* JK2 and JK3 indicated the inefficiency of KanJ and KanK. Thus, an increase in KanJK expression may enhance their total activity and gentamicin B production. The P*hrd*B promoter of *Streptomyces coelicolor*is stronger than P*erm*E* [20]. Therefore, a *kan*JK under the control of P*hrd*B was constructed and introduced to *M. echinospora* ∆K∆P; as a result, a mutant strain *M. echinospora* JK4 was generated (Additional file 1: Figure S1E).

The HPLC analysis of the fermentation broth of *M. echinospora* JK4 revealed that gentamicin B production reached 880 μg/ml; by contrast, JI-20A production decreased to 143 μg/ml. Figure 5b shows that gentamicin B production increased by 2.5-fold when the P*hrd*B promoter was used compared with that when P*erm*E* was used. This result confirmed that the expression level of KanJK was enhanced by replacing the promoter P*erm*E* with P*hrd*B.

The genome analysis of *Actinomyces* revealed the presence of numerous gene clusters encoding secondary metabolites [21]. *Actinomyces* can be metabolically engineered to overproduce native metabolites or analogs. With the development of synthetic biological tools, improved bioinformatics tools of metabolic engineering, and enhanced sensitivity and sophistication of analytical methods, secondary metabolite production is feasible in various cellular factories [22].

## Conclusions

We successfully established an artificial biosynthetic pathway to achieve a high-level production of gentamicin B. The genes *gen*K and *gen*P were disrupted in *M. echinospora* to producethe JI-20A, which is the precursor of gentamicin B. JI-20Aproduction in the gene-disrupting strain *M. echinospora* ΔKΔP reached 911 μg/ml, which was 14-fold higher than that of *M. echinospora* ∆P. We disrupted the biosynthesis of gentamicin C1, C1a, C2, C2a, JI-20B, and JI-20Ba by disrupting *gen*P and *gen*K. The removal of byproducts in fermentation broth will be beneficial to purification because antibiotic purification is costly and time consuming.

An artificial pathway for the conversion of JI-20A to gentamicin B was constructed through the heterologous overexpression of *kan*J and *kan*K in *M. echinospora* ΔKΔP. The *kan*JK-overexpressing strain under the control of PermE* can produce 80 μg/ml gentamicin B, which was tenfold higher than that of the wild-type strain. Different promoters and gene integration combinations were investigated to improve gentamicin B production. When the P*hrd*B promoter was used and *kan*J and *kan*K were integrated in the genome through gene replacement, gentamicin B was produced as a major product with a maximum yield of 880 μg/ml.These results confirmed that microbiological strains can be engineered through the metabolic engineering of an intrinsic biosynthetic pathway and the introduction of exogenous genes to produce high yields of target products.

## Methods

### Bacterial strains, plasmids, media, and culture conditions

The strains and plasmids used in this work are listed in Table 2. *Escherichia coli* Top10 was used as the cloning host grown on Luria–Bertani (LB) liquid or solid medium. Liquid ATCC172 was used for the vegetative growth of *M. echinospora*. The conjugal transfer was performed on MS agar. Solid slanting medium was used for *M. echinospora* sporulation. The previously described media and culture conditions were used for gentamicin production [8].

**Table 2:** Strains and plasmids used in this study

Strains or plasmids	Relevant characteristic	Reference or source
Strains		
*E. coli* TOP10	F-*mcr*AΔ(*mrr*-*hsd*RMS-*mcr*BC), φ80*lac*ZΔM15,Δ*lac*X74, *deo*R, *rec*A1, *ara*D139Δ(*ara*-*leu*)7697, *gal*U, *gal*K, *rps*L(Str^R^), *end*A1, *nup*G	Invitrogen
*E. coli* ET12567/pUZ8002	Methylation defective, strain used in *E. coli*-*streptomyces* intergeneric conjugation	[25]
*S. kanamyceticus*	Kanamycin producing strain	CGMCC4.1441
*M. echinospora*	Wild-type strain, gentamicin C1a, C2, C2a, and C1 producer	ATCC 15835
*M. echinospora*ΔP	*M. echinospora* with disrupted *gen*P	[7]
*M. echinospora*ΔKΔP	*M. echinospora* with disrupted *gen*K and *gen*P	This study
*M. echinospora* JK1	Heterologous, genome-based expression of *kan*J and *kan*K, with replacement of the native promoter by P*erm*E* in *M. echinospora*ΔKΔP.	This study
*M. echinospora* JK2	*M. echinospora*ΔKΔP + heterologous expression of *kan*J and *kan*K under the promoter P*erm*E*, insertion at *gen*P locus in *M. echinospora* ΔKΔP	This study
*M. echinospora* JK3	Heterologous expression of *kan*J and *kan*K under promoter P*gen*P, insertion at *gen*P locus in *M. echinospora* ΔKΔP	This study
*M. echinospora* JK4	Heterologous expression of *kan*J and *kan*K under promoter P*hrd*B, insertion at *gen*P locus in *M. echinospora* ΔKΔP	This study
Plasmids		
pKC1139	*E. coli*-*streptomyces* shuttle vector, Am^R^	[26]
pSPU241	pIJ2925 derivative carrying the *Streptomyces* constitutive promoter P*erm*E* and To terminator, Amp^R^	[8]
pEAP1	pSET152 carrying *erm*E, the apramycin resistance-conferring gene *aac*(3)IV was replaced by the ampicillin resistance-conferring gene *bla*, Amp^R^, Erm^R^	[8]
pSPU503	pKC1139 carrying homologous arms of genK (*gac*D), used in *gen*K disruption	[12]
pJK1	pEAP1 carrying P*erm*E*-*kan*J-*kan*K, used in generating *M. echinospora* JK1	This study
pJK2	pKC1139 carrying homologous arms and *kan*JK, used in generating *M. echinospora* JK2	This study
pJK3	pKC1139 carrying homologous arms and P*erm*E*-*kan*J-*kan*K, used in generating *M. echinospora* JK3	This study
pJK4	pKC1139 carrying homologous arms andP*hrd*B-*kan*J-*kan*K, used in generating *M. echinospora* JK4	This study

*Amp* ^*R*^ ampicillin resistance, *Am* ^*R*^ ampramycin resistance, *Erm* ^*R*^ erythromycin resistance

### Construction of *kan*J and *kan*K expression plasmids

DNA isolation and manipulation were performed as described by Sambrook [23]. Additional file 3: Table S1 lists the primers used in this work. The *kan*J and *kan*K genes were amplified from the genomic DNA of *S. kanamyceticus*. The primers were designed using the biosynthetic gene sequence of kanamycin (GenBank accession number: AJ628422.2) and gentamicin (GenBank accession number:AJ628149.4). The primers Pkanjk-up1 and Pkanjk-down1 were used to amplify a 1.95 kb fragment containing intact *kan*J and *kan*K. The PCR product was digested with *Hin*dIII and *Bam*HI and then ligated to pSPU241; thus, pJK241 was generated. The 2.6 kb insert containing P*erm*E*-*kan*JK-To was recovered as a *Bgl*II fragment and then inserted into the same site of pEAP1 to generate pSPUJK1.

The primers Ph1 and Ph2 were used to amplify the downstream homologous arm and to generate a gene replacement vector with *kan*JK genes under P*erm*E*. The primers Ph3 and Ph4 were utilized to amplify the upstream homologous arm. Pkanjk-up2 and Pkanjk-down2 were also employed to amplify the intact *kan*JK fragment. Perm-up and Perm-down were used to amplify P*erm*E*. P*erm*E*, *kan*JK, and the upstream homologous arm fragment were fused through overlap extension PCR in accordance with a previously described procedure [24]. The overlapping PCR product was ligated with pMD18-T (Takara) and digested with *Xba*I and *Sma*I; the downstream homologous arm was digested with *Xba*I and *Sma*I. Afterward, the digested fragments were ligated to pKC1139; thus, pSPUJK2 was generated.

The primers Ph1 and Ph2 were used to amplify the downstream homologous arm and the primers Ph33 and Ph4 were utilized to amplify the upstream homologous arm to generate a vector for gene replacement. Pkanjk-up3 and Pkanjk-down2 were also used to amplify *kan*JK. The overlap extension PCR was employed using the primers Ph4 and Pkanjk-down2 to fuse the *kan*JK fragment and the upstream homologous arm. The overlap PCR product was ligated using pMD18-T(Takara) and then digested with *Xba*I and *Sma*I. The downstream homologous arm was digested with *Xba*I and *Sma*I. The digested fragments were then ligated to pKC1139; thus, pSPUJK3 was generated.

The primers Ph34 and Ph4 were used to amplify the upstream homologous arm to generate a gene replacement vector with *kan*JK genes under the strong promoter P*hrd*B. Pkanjk-up4 and Pkanjk-down2 were utilized to amplify the intact *kan*JK fragment. Phrd-up and Phrd-down were also used to amplify P*hrd*B. The upstream homologous arm fragment, P*hrd*B, and *kan*JK were then fused through overlap extension PCR. The overlap PCR product was then ligated with pMD18-T and then digested with *Xba*I and *Sma*I. The downstream homologous arm was digested with *Xba*I and *Sma*I. The digested fragments were ligated to pKC1139. Thus, pSPUJK4 was generated.

### Construction of *gen*K-disrupting and *kan*JK-expressing strains

*gen*K-disrupting and *kan*JK-expressing plasmids were introduced to *M. echinospora* through conjugation on MS medium at 28 °C for 24 h. After the medium was spread with 50 mg of apramycin (erythromycin was used for pSPUJK1) and 100 mg of pipemidic acid per liter, incubation was performed at 28 °C for 7 days. Exconjugants were initially selected to determine the apramycin-resistance (the first crossover event) phenotype because pSPU503, pSPUJK2, pSPUJK3, and pSPUJK4 plasmids contain an apramycin-resistance gene; the apramycin-sensitive (the second crossover event) phenotype was then used to isolate the strains via the desired double-crossover homologous recombination event. Genomic DNA was extracted and used as a template DNA in PCR. The PCR products were subjected to DNA sequencing to demonstrate that *kan*J and *kan*K exist in the *kan*JK-expressing strains.

### Antibiotic isolation and analysis

The pH of the culture broth was adjusted to 2.0 by using H_2_SO_4_. The acidified broth was agitated for 30 min and then centrifuged at 11,378×*g* 10 min. The pH of the supernatant was readjusted to 7.0 with NaOH. The pretreated supernatant was centrifuged again at 11,378×*g* for 10 min. The supernatant was then applied to strongly acidic resin 001 × 7 (Shandong Lukang Record Pharmaceutical Co., Ltd).The bound substances were eluted with 2 mol/L NH_4_OH. Second cation-exchange chromatography was performed on weakly acidic resin D152 (Shandong Lukang Record Pharmaceutical Co., Ltd). The bound substances were eluted with the gradient elution of NH_4_OH (from 0.1 to 1.0 mol/L).

The elution from the acidic resin was used as the sample for the reversed-phase HPLC-ELSD analysis in a reverse C18 column at an evaporation temperature of 45 °C, nitrogen pressure of 3.5 bar, and a mobile phase of 0.2 mol/L trifluoroacetatic acid–methanol (97:3) at a flow rate of 0.6 ml/min. Authentic gentamicin B was used as standard. The purified products were analyzed using an LC/MS/MS instrument (Bruker micrOTOF-Q). The mass spectrometer was set in a positive mode. ^1^H and ^13^C NMR data were recorded on Bruker AV600 at 600 MHz frequency, and D_2_O was used as a solvent.

## Additional file

10.1186/s12934-015-0402-6 Schematic representation and confirmation of recombination strains. (A) Gene disruption of *gen*K and *gen*P. (B) Expression of *kan*J and *kan*K by site specific intergration. (C) Expression of *kan*J and *kan*K with the promoter *Perm*E*. (D) Expression of *kan*J and *kan*K with the native promoter of genP. (E) Expression of *kan*J and *kan*K with promoter *Phrd*B.
10.1186/s12934-015-0402-6 ^1^H NMR spectrum of the new compound from *kan*JK expression strains.
10.1186/s12934-015-0402-6 Sequence of primers used in this study.
