# Supplementary material for: Assembly of a novel biosynthetic pathway for gentamicin B production in Micromonospora echinospora
Source: Microb Cell Fact. 2016 Jan 5;15:1. doi: 10.1186/s12934-015-0402-6 (PMC4700567; doi:10.1186/s12934-015-0402-6)

Additional file 1: Figure S1. Schematic representation and confirmation of recombination strains. (A) Gene disruption of *gen*K and *gen*P (B) Expression of *kan*J and *kan*K by site specific intergration. (C) Expression of *kan*J and *kan*K with the promoter P*erm*E* (D) Expression of *kan*J and *kan*K with the native promoter of *gen*P (E) Expression of *kan*J and *kan*K with promoter P*hrd*B.

Figure S1 Schematic representation and confirmation of recombination strains. (A) Gene disruption of *gen*K and *gen*P

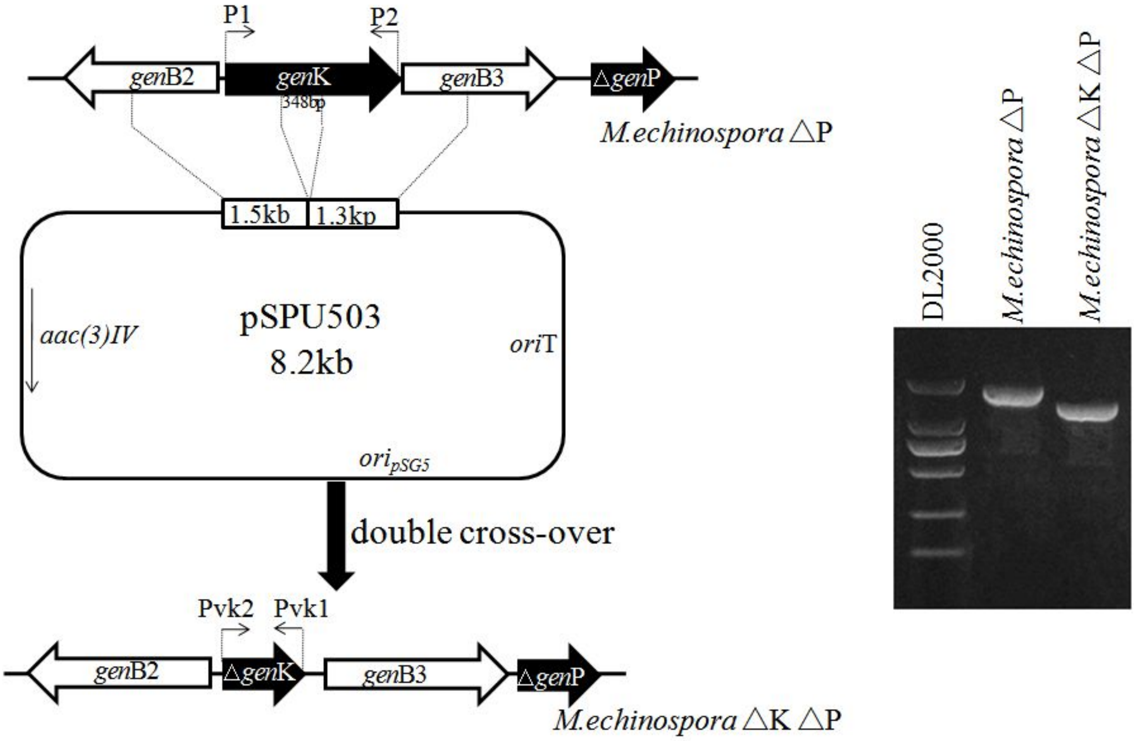

Figure S1 Schematic representation and confirmation of recombination strains. (B) Expression of *kan*J and *kan*K by site specific intergration.

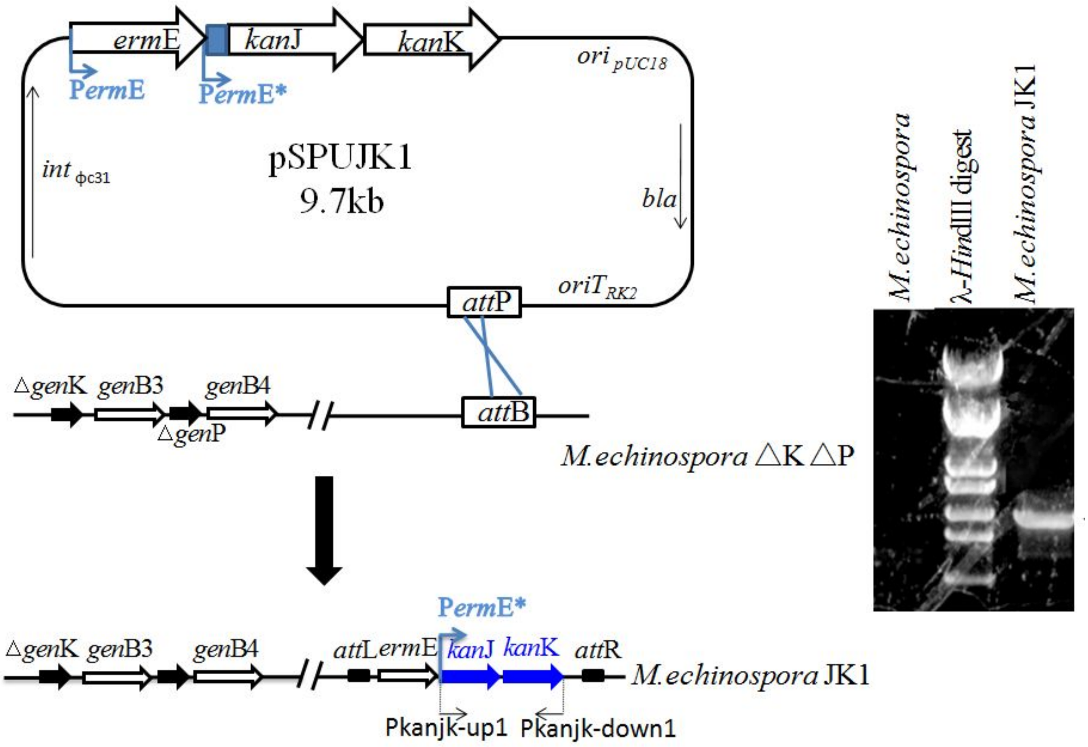

Figure S1 Schematic representation and confirmation of recombination strains. (C) Expression of *kan*J and *kan*K with the promoter P*erm*E*.

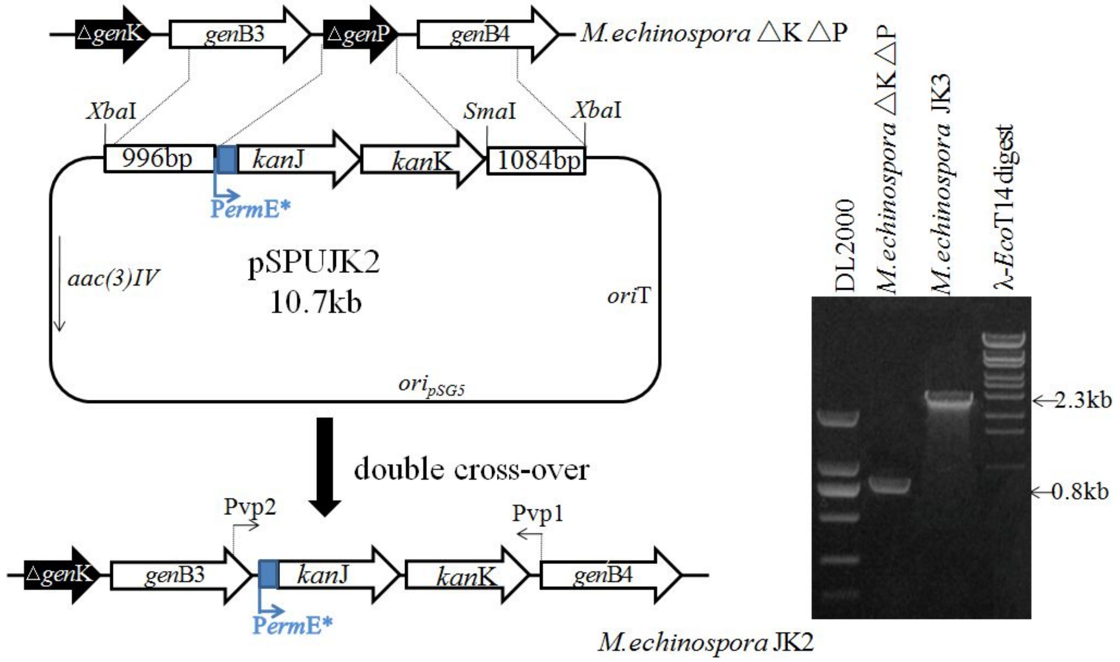

Figure S1 Schematic representation and confirmation of recombination strains. (D) Expression of *kan*J and *kan*K with the native promoter of *gen*P.

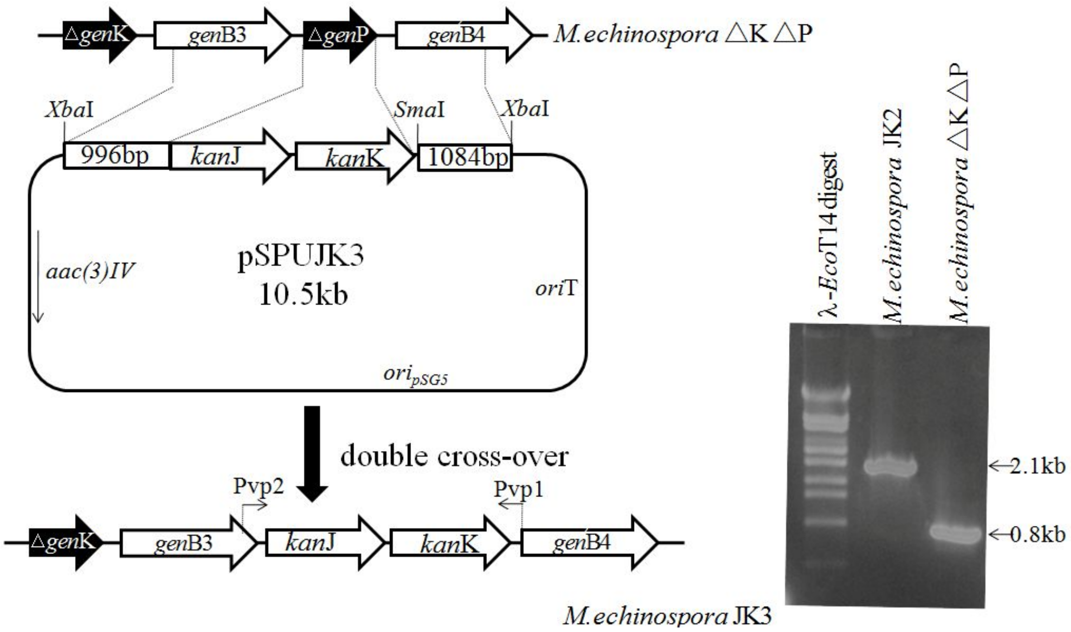

Figure S1 Schematic representation and confirmation of recombination strains. (E) Expression of *kan*J and *kan*K with promoter P*hrd*B.

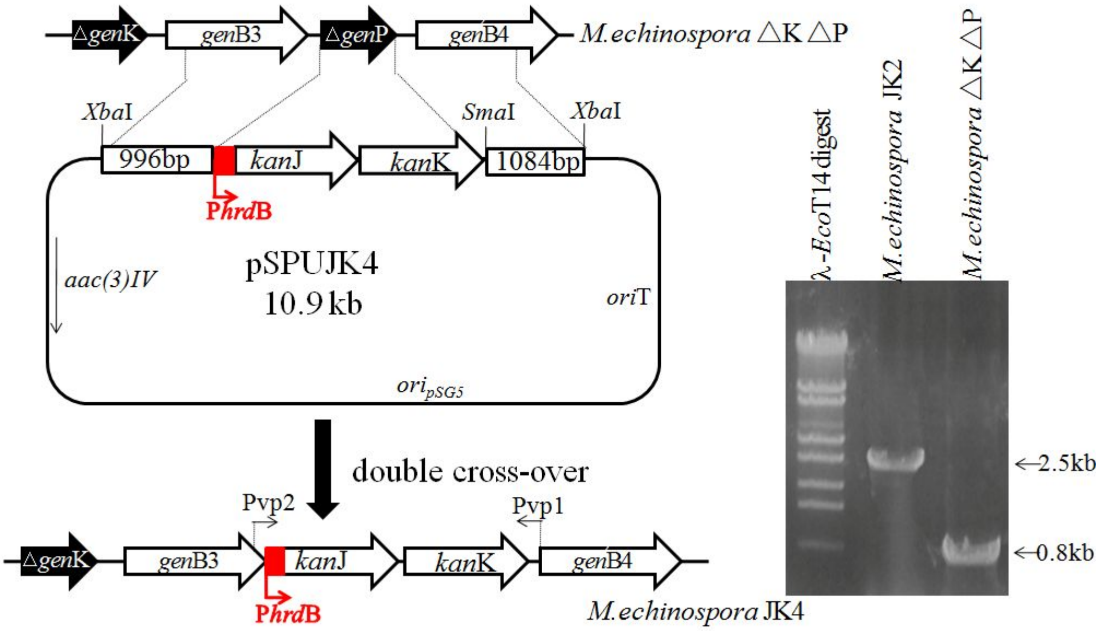

Supplement: Supplementary file 1 — 10.1186/s12934-015-0402-6 Schematic representation and confirmation of recombination strains. (A) Gene disruption of genK and genP. (B) Expression of kanJ and kanK by site specific intergration. (C) Expression of kanJ and kanK with the promoter PermE*. (D) Expression of kanJ and kanK with the native promoter of genP. (E) Expression of kanJ and kanK with promoter PhrdB. [file 12934_2015_402_MOESM1_ESM.pdf]
