# Supplementary material for: Assembly of a novel biosynthetic pathway for gentamicin B production in Micromonospora echinospora
Source: Microb Cell Fact. 2016 Jan 5;15:1. doi: 10.1186/s12934-015-0402-6 (PMC4700567; doi:10.1186/s12934-015-0402-6)

Additional file 2: Figure S2. ^{1}H NMR spectrum of the new compound from *kan*JK expression strains.

Figure S2 ^{1}H NMR spectrum of the new compound from *kan*JK expression strains.

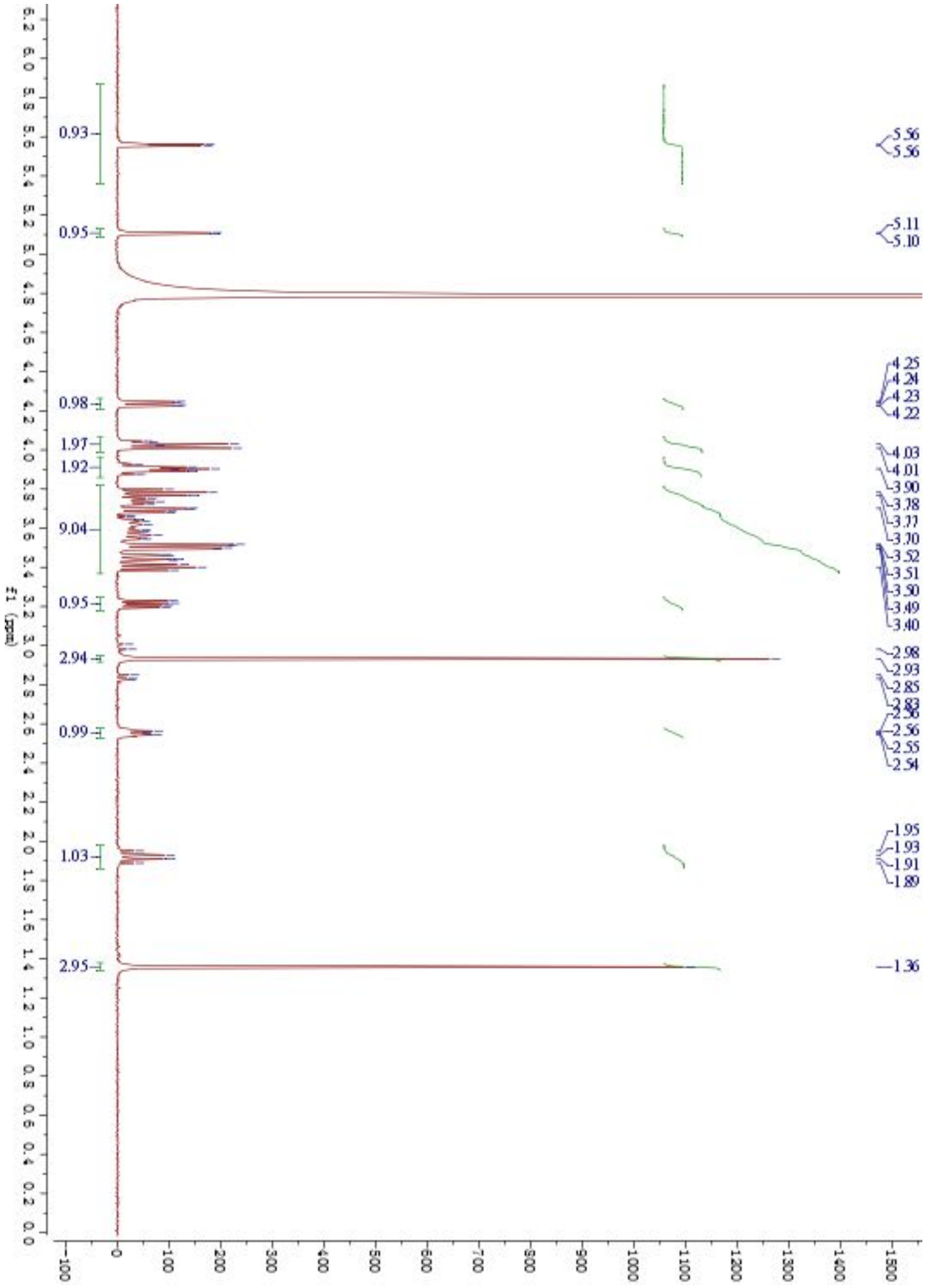

Supplement: Supplementary file 2 — 10.1186/s12934-015-0402-6 1H NMR spectrum of the new compound from kanJK expression strains. [file 12934_2015_402_MOESM2_ESM.pdf]
