# Supplementary material for: Assembly of a novel biosynthetic pathway for gentamicin B production in Micromonospora echinospora
Source: Microb Cell Fact. 2016 Jan 5;15:1. doi: 10.1186/s12934-015-0402-6 (PMC4700567; doi:10.1186/s12934-015-0402-6)
Supplement: Supplementary file 3 — 10.1186/s12934-015-0402-6 Sequence of primers used in this study. [file 12934_2015_402_MOESM3_ESM.pdf]

Additional file 3: Table S1. Sequence of primers used in this study.

Primers	Sequence
Ph1	5'-GCTCTAGATGCGCCGTCTTGTAGATCTCC-3'
Ph2	5'-TCCCCCGGGCGGCGACCGACGCCGG-3'
Ph3	5'-CGGACACTCGCATGGACGTCCTTCACCGGCCCTGTCGTTC-3'
Ph4	5'-GCTCTAGAGCTGGCACGAGTGGCAGTTGGCG-3'
Ph33	5'-GGGCGGAGCGGCAAGGGCCATCTTCACCGGCCCTGTCGTTC-3'
Ph34	CGTTCCGGCGGAAGGCGGAATTCCTTCACCGGCCCTGTCGTTC
Pkanjk-up1	5'-TTTGGATCCAGCGATGGCCCTTGCCGCTCC-3'
Pkanjk-down1	5'-GCGAAGCTTCATCGGCCGAAATCACACCAG-3'
Pkanjk-up2	5'-CCGGTTGGTAGGATCCAGCGATGGCCCTTGCCGCTCCGCCC-3'
Pkanjk-down2	5'-TCCCCCGGGTCCAGTCACACCAGCCCTGGCGCGGTG-3'
Pkanjk-up3	5'-GAACGACAGGGCCGGTGAAGATGGCCCTTGCCGCTCCGCCC-3'
Pkanjk-up4	5'-CAACGTTCCGAGAGGTTGTCCATGGATGGCCCTTGCCGCTCCGCC-3'
Perm-down	5'-GGGCGGAGCGGCAAGGGCCATCGCTGGATCCTACCAACCGG-3'
Perm-up	5'-GAACGACAGGGCCGGTGAAGGACGTCCATGCGAGTGTCCG-3'
PhrdB-up	5'-GAACGACAGGGCCGGTGAAGGAATTCCGCCTTCCGCCGGAACG-3'
PhrdB-down	5'-GGCGGAGCGGCAAGGGCCATCCATGGACAACCTCTCGGAACGTTG-3'
Pvk1	5'-AAGCTTTGGGACGAGGGTCAGTGGGA-3'
Pvk2	5'-CATATGATGAACGCGCTGGTGGCAG-3'
Pvp1	5'-TGTGCCCACCACGCCATTC-3'
Pvp2	5'-GCAGCATCAAGCCGGTTCTCA-3'
